# Regulation of Phosphoinositide Levels in the Retina by Protein Tyrosine Phosphatase 1B and Growth Factor Receptor-Bound Protein 14

**DOI:** 10.3390/biom11040602

**Published:** 2021-04-19

**Authors:** Raju V. S. Rajala, Austin McCauley, Rahul Rajala, Kenneth Teel, Ammaji Rajala

**Affiliations:** 1 Department of Ophthalmology, University of Oklahoma Health Sciences Center, Oklahoma City, OK 73104, USA; austin-mccauley@ouhsc.edu (A.M.); kenneth-teel@ouhsc.edu (K.T.); ammaji-rajala@ouhsc.edu (A.R.); 2 Department of Physiology, University of Oklahoma Health Sciences Center, Oklahoma City, OK 73104, USA; 3 Department of Cell Biology, University of Oklahoma Health Sciences Center, Oklahoma City, OK 73104, USA; rahul-rajala@omrf.org; 4 Dean McGee Eye Institute, Oklahoma City, OK 73104, USA; 5 Cardiovascular Biology Program, Oklahoma Medical Research Foundation, Oklahoma City, OK 73104, USA

**Keywords:** phosphoinositides, retina, photoreceptor cells, membrane binding, light activation, PTP1B, Grb14

## Abstract

Protein tyrosine kinases and protein phosphatases play a critical role in cellular regulation. The length of a cellular response depends on the interplay between activating protein kinases and deactivating protein phosphatases. Protein tyrosine phosphatase 1B (PTP1B) and growth factor receptor-bound protein 14 (Grb14) are negative regulators of receptor tyrosine kinases. However, in the retina, we have previously shown that PTP1B inactivates insulin receptor signaling, whereas phosphorylated Grb14 inhibits PTP1B activity. In silico docking of phosphorylated Grb14 and PTP1B indicate critical residues in PTP1B that may mediate the interaction. Phosphoinositides (PIPs) are acidic lipids and minor constituents in the cell that play an important role in cellular processes. Their levels are regulated by growth factor signaling. Using phosphoinositide binding protein probes, we observed increased levels of PI(3)P, PI(4)P, PI(3,4)P_2_, PI(4,5)P2, and PI(3,4,5)P_3_ in PTP1B knockout mouse retina and decreased levels of these PIPs in Grb14 knockout mouse retina. These observations suggest that the interplay between PTP1B and Grb14 can regulate PIP metabolism.

## 1. Introduction

Protein tyrosine phosphatase 1B (PTP1B) and growth factor receptor-bound protein (Grb14) are two negative regulators of insulin receptor (IR) and insulin-like growth factor 1 receptor (IGF-1R) [1,2]. We previously reported that PTP1B and Grb14 are expressed in various layers of the retina, including photoreceptor cells, where IR and IGF-1R are expressed [3]. Furthermore, we noted increased PTP1B activity in the dark-adapted retina, and decreased PTP1B activity was observed in the light-adapted conditions [4]. PTP1B belongs to the protein tyrosine phosphatase family, dephosphorylates insulin and IGF1 receptors, and inactivates IR and IGF1R signaling [1]. Either conditional deletion of PTP1B in rods or global PTP1B deletion resulted in enhanced retinal neuroprotection [4,5], whereas mouse rods lacking insulin receptors are susceptible to light-induced photoreceptor degeneration [6]. In cones, deletion of IR resulted in cone degeneration in the absence of stress [7].

Grb14 is a pseudosubstrate inhibitor of the IR, which interacts with IR’s substrate binding site and selectively inhibits the IR kinase activity [2]. Tissue-specific effects have been observed when mice lack Grb14. Increased insulin sensitivity and enhanced glucose tolerance have been observed in the liver and skeletal muscle of Grb14 KO mice [8]. However, in the liver, a decreased IR phosphorylation has been observed as a result of increased protein tyrosine phosphatase activity [8]. In the retina, loss of Grb14 resulted in the loss of light-induced tyrosine phosphorylation of the IR [3]. In cardiac muscle, ablation of Grb14 led to decreased phosphoinositide 3-kinase (PI3K)/Akt activation and myocardial infarction [9]. The retina is a post-mitotic tissue, and downregulation of growth factor signaling is detrimental to retinal neurons. Both PTP1B and Grb14 negatively regulate insulin and IGF1R signaling. How do the retinal IR and IGF1R overcome the inhibition by PTP1B and Grb14? We previously reported that Grb14 undergoes tyrosine phosphorylation by a non-receptor tyrosine kinase, Src, and the phosphorylated Grb14 competitively inhibits PTP1B [10]. Upon PTP1B inhibition, the IR and IGF1 receptors activate the downstream PI3K/Akt activation.

**Figure 1:**
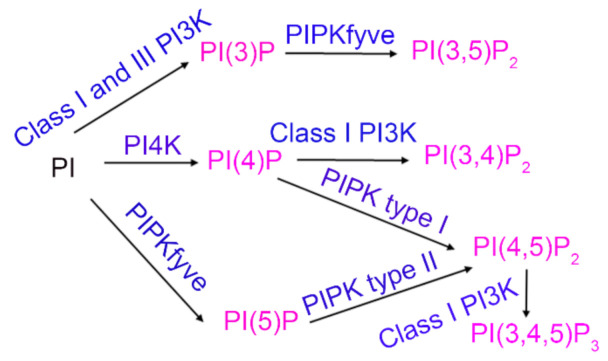
Generation of seven phosphoinositides. Phosphatidylinositol (PI) undergoes phosphorylation at 3, 4, 5-free hydroxyl groups and generates seven distinct PIPs. Class I phosphoinositide 3-kinase (PI3K) phosphorylates PI to PI(3)P, PI(4)P to PI(3,4)P_2,_ and PI(4,5)P_2_ to PI(3,4,5)P_3_. Phosphoinositide 4-kinase (PI4K) phosphorylates PI to PI(4)P. Phosphatidylinositol 3-phosphate 5-kinase (PIKfyve) phosphorylates PI to PI(5)P and PI(3)P to PI(3,5)P_2_. Type I phosphatidylinositol 4-phosphate 5-kinase (PIPKI) phosphorylates PI(4)P to PI(4,5)P_2_. Type II phosphatidylinositol 5-phosphate 4-kinase (PIPKII) phosphorylates PI(5)P to PI(4,5)P_2_. Pink, PIPs; Blue, PIP-kinases.

Phosphoinositide lipids (also known as phosphatidylinositol phosphates or PIPs) are minor constitutes of phospholipids [11]. Phosphoinositides are generated by the parent molecule phosphatidylinositol, which consists of a myo-inositol head group, a glycerol backbone, and two fatty acids attached to the C1 and C2 position of glycerol [12]. Phosphorylation of free-hydroxyl on 3, 4, 5 positions on the inositol ring by phosphoinositide kinases resulted in the generation of seven phosphorylated phosphoinositides (PIPs): PI(3)P, PI(4)P, PI(5)P, PI(3,4)P_2_, PI(3,5)P_2_, PI(4,5)P_2_, and PI(3,4,5)P_3_ (Figure 1). These PIPs play an important role in membrane budding and fusion, ciliogenesis, cytoskeletal assembly, vesicular trafficking, and signal transduction [11]. In the present study, we examined the levels of phosphoinositide lipids in PTP1B and Grb14 KO mice. 

## 2. Materials and Methods

### 2.1. Materials

Polyclonal Grb14 antibody was generated as described [3]. Polyclonal anti-PTP1B antibody was obtained from Upstate Biotechnology (Lake Placid, NY, USA). The actin antibody was obtained from Affinity BioReagents (Golden, CO, USA). All other reagents were of analytical grade and from Millipore Sigma (St Louis, MO, USA).

### 2.2. Animals

We followed the National Institute of Health (NIH) Guide for the Care and Use of Laboratory Animals and ARVO Statement for the Use of Animals in Ophthalmic and Vision Research. The Institutional Animal Care and Use Committee (IACUC) at the University of Oklahoma Health Sciences Center approved all protocols (Protocol #13-027). PTP1B KO mice were obtained from Dr. Benjamin Neel (Harvard Medical School). We characterized these mice in our previous publication [4]. Grb14 KO mice were a kind gift from Roger J. Daly (Garvan Medical Institute, Sydney, Australia). We characterized these mice in our previous publications [3,10]. Retinas were harvested after CO_2_ asphyxiation and were used for immunoblot and phosphoinositide analyses. 

### 2.3. Methods

#### 2.3.1. Extraction and Measurement of Phosphoinositides from the Retina

Mouse retinas were homogenized in phosphate-buffered saline (PBS) and the retinal phosphoinositides were extracted as described [13]. Lipid phosphorous was measured according to the method described [14]. Phosphoinositide lipids PI(3)P, PI(4)P, PI(3,4)P_2_, PI(4,5)P_2_, and PI(3,4,5)P_3_ were measured by ELISA using PIP-binding protein probes [13]. The obtained luminescence was normalized to phospholipid [15], calculated by measuring lipid phosphorous [14] in the extracted phosphoinositide fraction from the respective retina. We used five mice per group (10 retinas) for the determination PIP levels. Student “t” test was used to compared the significne between the wild-type and KO mouse retinas.

#### 2.3.2. Immunoblot Analysis

Immunoblot analysis was carried out as described [3]. The blots were probed with anti-PTP1B (1:1000), anti-Grb14 (1:1000), and anti-actin (1:1000) antibodies overnight at 4 °C. Following overnight incubation in primary antibodies, blots were incubated in either HRP-linked anti-rabbit or anti-mouse secondary antibodies and developed by ECL reagent.

#### 2.3.3. In Silico Modeling of Grb14 and PTP1B Interactions

FASTA sequences for mouse Grb14 (Q9JLM9) and mouse PTP1B (P35821) were isolated from the UniProt Repository. Sequences were then processed using the SWISS-Model [16,17], and using the PTP1B template (1SUG) [18] and Grb14 template (4K81) [19], we generated molecular models of proteins. The programs AutoDock Tools and AutoDock VINA [20] were used to perform docking simulations of the C-terminal tail of Grb14 from amino acids 341–354 [10] as well as phosphorylated Grb14 (pGrb14) peptide from residues 345–352 [10] with the PTP1B binding site. Phosphorylation was performed on Tyr (345, 348, 352) in silico using the PyMOL plugin PyTMs [21]. The Grb14-PTP1B binding site was estimated to occupy the same region as the PTP1B with the IR binding site (RCSB: 1G1H). The PTP1B binding site was visualized and circumscribed with a grid. Constraints are as follows (center_x = 39.995, center_y = 15.377, center_z = 11.841, size_x = 20, size_y = 20, size_z = 10, exhaustiveness = 8). Docking was performed with both the unphosphorylated and phosphorylated Grb14 ligand.

Target Preparation: The modeled structure of PTP1B from SWISS-Model was opened in AutoDock Tools. Polar hydrogens were added to PTP1B. The structure was then saved as a pdbqt file using AutoDock Tools. 

Ligand Preparation: The Grb14 structure from SWISS-Model was opened in PyMOL and the C-terminal tail of Grb14 was saved as a separate molecule. The PyMOL plugin PyTMs was used to add phosphates to Tyr (345, 348, 352); the resulting pGrb14 and Grb14 peptides were saved as PDB files. The pGrb14 and Grb14 peptides were individually opened in AutoDock Tools; the torsion count was modified so that the peptide backbone bonds in the Grb14 ligand were non-rotatable, but pGrb14 was rotatable. This was due to desolvation penalties associated with the presence of phosphate groups on alpha-helices [22]. Therefore, we opted to allow Vina to postulate the optimal conformation of the pGrb14 with PTP1B by lowering torsional constraints. The resulting ligands were saved as pdbqt files and docked to PTP1B using AutoDock Vina.

## 3. Results

### 3.1. Phosphoinositide Levels in PTP1B and Grb14 KO Mouse Retina

**Figure 2:**
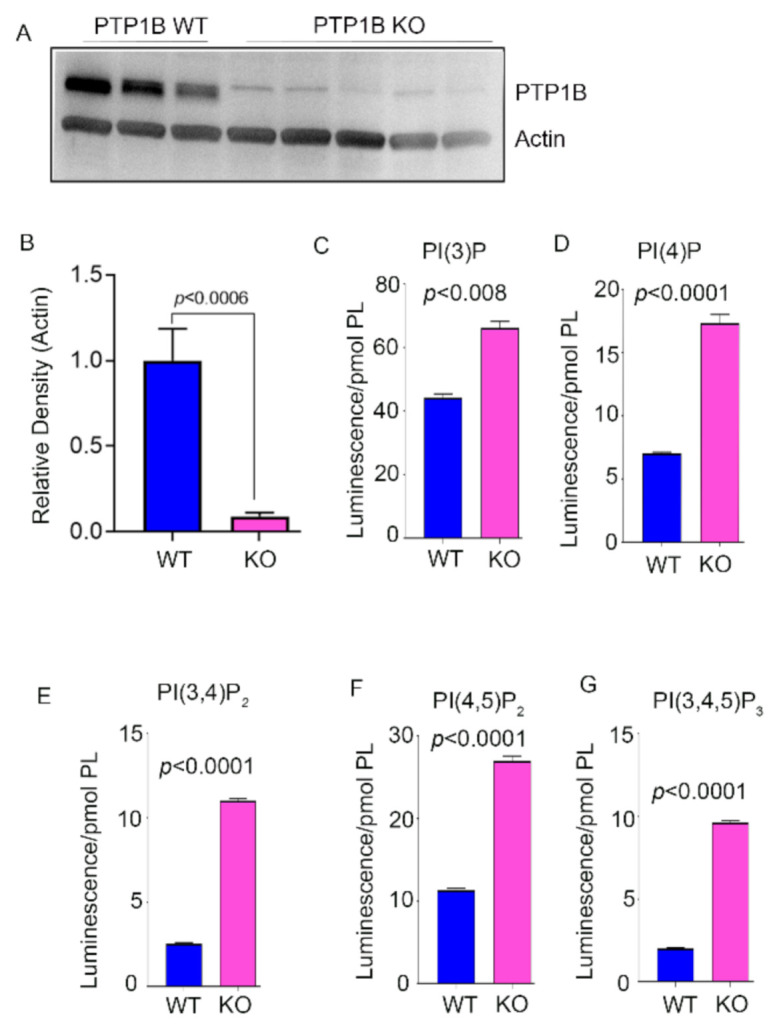
Determination of the levels of PIPs in the PTP1B knockout mouse retina. Wild-type and PTP1B KO mouse retina lysates were immunoblotted with anti PTP1B and anti-actin (**A**) antibodies. Densitometric analysis of PTP1B was normalized to actin (**B**). Data are mean ± *SEM*, (WT, *n* = 4; KO, *n* = 5). Student t-test was used to compare the significance between groups. Phosphoinositides were extracted from PTP1B KO and wild-type control littermates. The PIP lipids were coated on the ELISA plate and we measured PI(3)P, PI(4)P, PI(3,4)P_2_, PI(4,5)P_2_, and PI(3,4,5)P_3_ levels using PI-binding proteins as probes (**B**–**G**). Data are mean ± *SEM* (*n* = 3). The significance is indicated on each panel. Full-length blots are presented in Supplementary Information (Figure S1).

**Figure 3:**
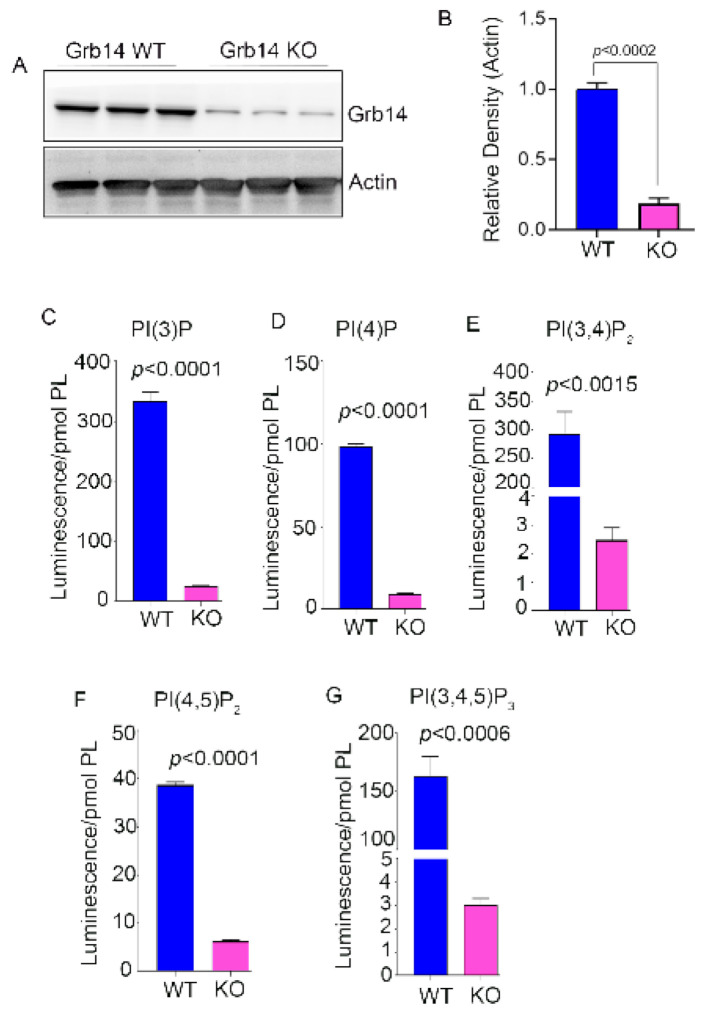
Determination of the levels of PIPs in the Grb14 knockout mouse retina. Wild-type and Grb14 KO mouse retina lysates were immunoblotted with anti Grb14 and anti-actin (**A**) antibodies. Densitometric analysis of Grb14 was normalized to actin (**B**). Data are mean ± *SEM*, (WT, *n* = 4; KO, *n* = 5). Student t-test was used to compare the significance between groups. Phosphoinositides were extracted from Grb14 KO and wild-type control littermates. The PIP lipids were coated on the ELISA plate and we measured PI(3)P, PI(4)P, PI(3,4)P_2_, PI(4,5)P_2_, and PI(3,4,5)P_3_ levels using PI-binding proteins as probes (**C**–**G**). Data are mean ± *SEM* (*n* = 3). The significance is indicated on each panel. Full-length blots are presented in Supplementary Information (Figure S1).

We characterized the PTP1B [4] and Grb14 KO [3,10] mice previously. Immunoblot analysis of eight-week-old wild-type and PTP1B KO mice indicated a loss of >98% of PTP1B expression in KO mice compared with wild-type mice (Figure 2A,B). We measured class I PI3K products PI(3)P, PI(3,4)P_2_, and PI(3,4,5)P_3_; class III PI3K product PI(3)P; PI4-kinase product PI(4)P; and PIPK1 type I and type II product PI(4,5)P_2_. We found significantly increased levels of PI(3)P, PI(4)P, PI(3,4)P_2_, PI(4,5)P_2_, and PI(3,4,5)P_3_ in PTP1B knockout mouse retinas compared with control littermates (Figure 2C–G). Immunoblot analysis of eight-week-old wild-type and Grb14 KO mice indicated a loss >81% of Grb14 in KO mice compared to wild-type mice (Figure 3A,B). We observed the residual Grb14 antibody immunoreactive band on immunoblots [23]. We previously reported that this band is not blocked by the blocking peptide [3]. Mouse retinas lacking Grb14 showed significantly decreased levels of PI(3)P, PI(4)P, PI(3,4)P_2_, PI(4,5)P_2_, and PI(3,4,5)P_3_ compared with wild-type littermates (Figure 3C–G).

### 3.2. Identification of Putative Residues in PTP1B That Mediate the Interaction with Grb14

**Figure 4:**
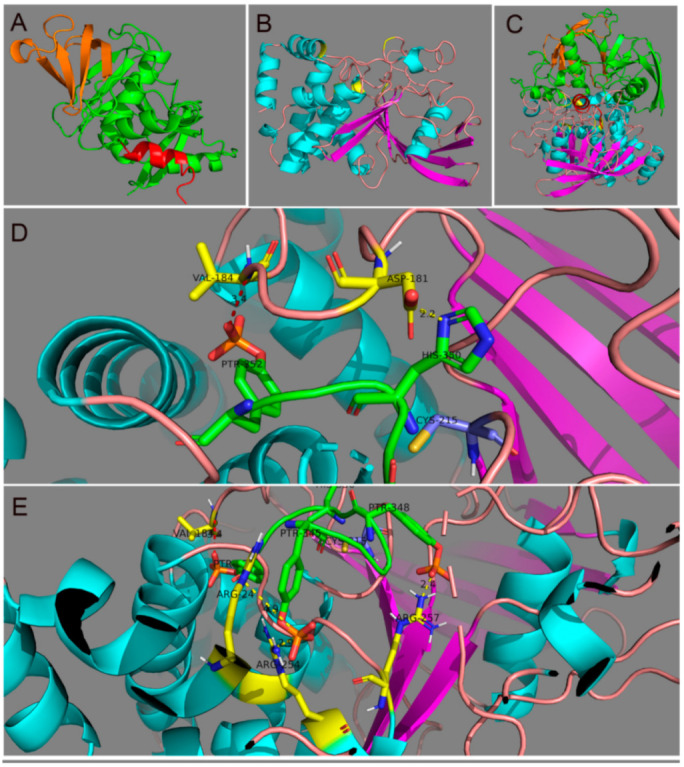
In silico modeling of Grb14 and PTP1B interactions. Cartoon model of Grb14. The binding peptide used for docking simulations is depicted in red. The PH domain is shown in orange (**A**). Cartoon model of PTP1B. The alpha helices are depicted in cyan. β-sheets are shown as purple. The predicted binding site for Grb14 is shown in yellow (**B**). A complex of Grb14 with PTP1B (**C**). The predicted binding interaction of the pGrb14 with PTP1B (pTyr352-Val-184; His350-Asp181) (**D**). The predicted binding interaction of the pGrb14 with PTP1B (pTyr345-Arg24; pTyr345-Arg254; pTyr348-Arg257) (**E**). Green, pGrb14 peptide; Yellow, putative interacting residues of PTP1B. Video files were provided in the Supplementary Information.

We have identified the minimum peptide region in Grb14 that is involved in the inhibition of PTP1B activity [10]. However, we have not studied the complementary interacting residues in PTP1B. In the present study, we used AutoDock Vina to identify binding interactions between PTP1B and Grb14 unphosphorylated and phosphorylated peptides. Note, due to the UniProt sequence, the residue count of Grb14 differs by two residues (e.g., Tyr347 becomes Tyr345) from that presented in our previously published paper [10]. Figure 4A shows the full-length Grb14 with docking peptide located at the C-terminus of the protein distal from the pleckstrin homology (PH) domain. Figure 4B shows the full-length PTP1B, and the predicted binding site of the Grb14 peptide is annotated. To show the full-length Grb14 in complex with PTP1B, the peptide was docked first and then the full-length protein was aligned to the peptide (Figure 4C, Supplementary Video 1). 

We previously reported that phosphorylated Grb14 (pGrb14) interacts with PTP1B, which results in the inhibition of its activity [10]. The docking analysis revealed possible interactions between pGrb14 and PTP1B that include a hydrogen bond between PTP1B-N-Val184 and pGrb14-pTyr352 (Figure 4D, Supplementary Video 2). We also found a salt bridge between PTP1B-Asp181 and pGrb14-H350 (Figure 4D, Supplementary Video 2). Prior studies have shown that the D181A mutant was able to interact with the substrate, but failed to dephosphorylate [24]. These observations suggest that H350 may interfere with the substrate accession by PTP1B. The pGrb14-pTyr348 interacts with PTP1B-Arg257 through a salt bridge (Figure 4E, Supplementary Video 2). The pGrb14-pTyr345 interacts with PTP1B-NE-Arg24 and PTP1B-Arg254 via salt bridges (Figure 4E, Supplementary Video 2).

## 4. Discussion

**Figure 5:**
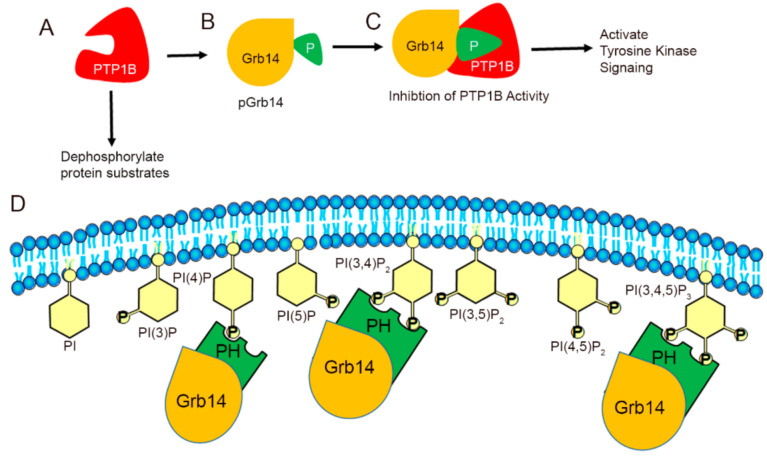
Mechanism of Grb14-mediated inhibition of PTP1B activity in the activation of the receptor tyrosine kinase signaling in the generation of PIPs. PTP1B dephosphorylates the protein substrates and inactivates the PI3K signaling (**A**). Grb14 undergoes phosphorylation (**B**) and the phosphorylated Grb14 binds PTP1B and inhibits its phosphatase activity (**C**), which results in the activation of the receptor tyrosine signaling, leading to the generation of PIPs (**C**). The interaction between PIPs and Grb14 (**D**).

We previously reported that PTP1B deleted mice exhibited enhanced retinal neuroprotection through constitutive activation of retinal insulin receptors [4]. In the retina, loss of Grb14 resulted in decreased IR phosphorylation due to increased PTP1B activity [3,10]. Our earlier studies showed that the non-phosphorylated form of Grb14 has a lower affinity for PTP1B than does the phosphorylated form of Grb14 [10] (Figure 5). Further, the in silico modeling predicted the possible interaction between Grb14 and PTP1B. The model predicted that pGrb14-Tyr345 interacts with PTP1B-Arg24 and Arg254 and pGrb14-pTyr348 interacts with PTP1B-Arg257 via salt bridges. Our model prediction also revealed the existence of a salt bridge between pGrb14-H350 and PTP1B-D181. The PTP1B-D181A mutant has previously been used as a substrate trap and identified several PTP1B substrates [24,25,26,27]. This mutant accesses the substrate but is unable to release and dephosphorylate the pTyr on the substrates [24]. Our prediction is that the interaction of pGrb14-H350 with PTP1B-D181 suggests a possible interference with the substrate in the inhibition of PTP1B activity.

An RMSD value of less than 2 Å may indicate the accuracy of the software. In AutoDock Vina, RMSD values are given by comparing multiple docking poses. However, the use of a macromolecule peptide generated only a few docking poses as opposed to a small ligand. Docking the unphosphorylated-Grb14 peptide gave only one docking pose (estimated affinity 281.6 kcal/mol), whereas the phosphorylated-Grb14 peptide gave two docking poses (estimated affinity 113.1/113.8 kcal/mol). The RMSD values between the docking pose for the phosphorylated Grb14 peptide was 3.9 Å. It has been well documented in the literature that peptides are more flexible than proteins and tend to adopt numerous conformations [28]. Consistent with the molecular modeling prediction, we previously reported that pGrb14 is a competitive inhibitor of PTP1B [10]. Our in silico model gives us a possible mechanistic insight into how pGrb14 acts as a competitive inhibitor of PTP1B. Further studies, however, are necessary to validate these predictions. 

In line with our earlier observations, loss of PTP1B resulted in increased PI(3)P, PI(4)P, PI(3,4)P_2_, PI(3,5)P_2_, and PI(3,4,5)P_3_, whereas loss of Grb14 decreased these PIPs in the retina. Together, these observations suggest that the interplay between PTP1B and Grb14 modulates the key enzymes involved in PIP metabolism. Insulin and IGF1 receptor activation are modulated by PTP1B and Grb14, which in turn regulate class I PI3K, which generates PI(3,4)P_2_ and PI(3,4,5)P_3._ The observed increase in PI(3,4)P_2_ and PI(3,4,5)P_3_ in PTP1B KO mouse retina and decreased levels of the PIPs in Grb14 KO mouse retinas suggest the negative and positive regulatory roles of PTP1B and Grb14 on insulin and IGF1 receptors or other tyrosine kinase receptors in the retina. Interestingly, we observed increased levels of PI(3)P, PI(4)P, and PI(4,5)P_2_ in PTP1B KO mouse retina. Decreased levels of these PIPs in the Grb14 KO mouse retina suggest that the key enzymes involved in the synthesis and degradation of PIPs may also be regulated by the interplay between PTP1B and Grb14. Further studies are needed to confirm these observations. The Grb14 has a PH domain, and we previously reported that it can bind to PIPs in vitro [29]. We speculate that the Grb14-PH domain interacts with membrane PIPs, whereas the C-terminus interacts with PTP1B, thereby promoting growth factor signaling (Figure 5).

Akt, a canonical pro-survival molecule downstream of PI3K, is constitutively active in cone photoreceptors [30]. The addition of the growth factor to retinal explants from rd1 mice resulted in significant activation of Akt [30]. We previously reported that the ablation of Akt2 in the retina resulted in stress-induced photoreceptor degeneration [31]. We also reported that loss of PI3K, which makes PI(3,4,5)P_3_ in cones, resulted in age-related cone degeneration [32]. Disruption of Akt signal transduction significantly contributes to the pathogenesis of various neurodegenerative diseases, such as Parkinson’s disease, Alzheimer’s disease, Huntington’s disease, and many others [33].

In the retina, there is an active PIP cycle that responds to light [12,34]. Both class I PI3K and class III P3K signaling pathways are essential for cone photoreceptor function and survival [32,35], whereas class III P3K is essential for rod photoreceptor [36], rod bipolar [37], and retinal pigment epithelium cell functions [38]. In the retina, PIPs play an important role in channel modulation, phototransduction, vesicular transport, ciliogenesis, and cell survival [12,34]. Furthermore, mutations in phosphoinositide phosphatases play an important role in retinal diseases. Mutations in synaptojanin 1 in zebrafish cone photoreceptors show an abnormal buildup of late endosomes and autophagosomes [39]. Defects in oculocerebroretinal syndromes of Lowe (OCRL) phosphatase are associated with glaucoma [40], and mutations in inositol polyphosphatase (INPP5E) are associated with Bardet-Bedi and Joubert syndrome, which leads to retinal degeneration [41,42]. The novel observations made on the interplay between PTP1B and Grb14 on PIPs in the present study suggest that other regulatory proteins may influence the PIP metabolism.

## 5. Conclusions

Phosphoinositide lipids play an important role in cellular processes. In cells, PIPs are formed by the action of phosphoinositide kinases and phosphoinositide phosphatases. Class I phosphoinositide 3-kinase activation via growth factor signaling enhances PI(3,4)P_2_ and PI (3,4,5)P_3_. In the current study, we showed that two regulators of the receptor tyrosine kinase signaling, PTP1B and Grb14, modulate the levels of PIPs in the retina. Our studies also showed modulations in the levels of PI(3)P, PI(4)P, and PI(4,5)P_2_ in PTP1B and Grb14 KO mouse retina, suggesting that the enzymes involved in the generation of these PIPs may also be influenced by the interplay between PTP1B and Grb14. Furthermore, our in silico docking of phosphorylated Grb14 and PTP1B indicate critical residues in PTP1B that may mediate the interaction.

## Supplementary Materials

The following are available online at https://www.mdpi.com/article/10.3390/biom11040602/s1, Figure S1: Full-length immunoblots, Video S1: docking of full-length Grb14 in complex with PTP1B, Video S2: docking of pGrb14 peptide with PTP1B.
