# Supplementary material for: Regulation of Phosphoinositide Levels in the Retina by Protein Tyrosine Phosphatase 1B and Growth Factor Receptor-Bound Protein 14
Source: Biomolecules. 2021 Apr 19;11(4):602. doi: 10.3390/biom11040602 (PMC8073254; doi:10.3390/biom11040602)
Supplement: Supplementary file 1 [file biomolecules-11-00602-s001.zip › Figure S1. Full-length immunoblot images.pdf]

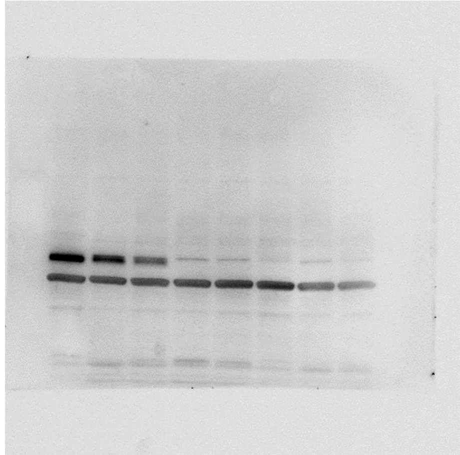

Full-length blot of Figure 2 (A) in the main text (PTP1B, top and actin, bottom).

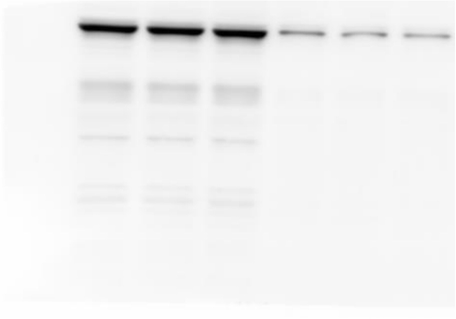

Full-length blot of Figure 3 (A) in the main text (Grb14).

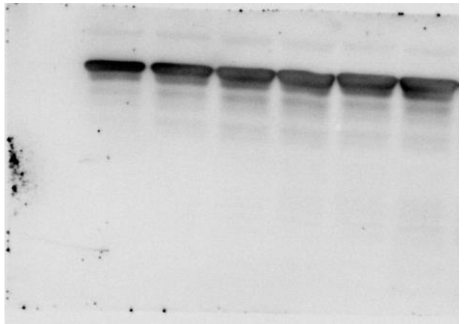

Full-length blot of Figure 3 (A) in the main text (actin).
